# Predictors of Return to Work 6 Months After the End of Treatment in Patients with Common Mental Disorders: A Cohort Study

**DOI:** 10.1007/s10926-017-9747-5

**Published:** 2017-12-12

**Authors:** Mattias Victor, Bjørn Lau, Torleif Ruud

**Affiliations:** 1 Lovisenberg Hospital, Nydalen, Postboks 4970, 0440 Oslo, Norway; 2 Institute of Clinical Medicine, University of Oslo, Oslo, Norway; 3 Department of Psychology, University of Oslo, Oslo, Norway; 4 Division Mental Health Services, Akershus University Hospital, 1478 Lørenskog, Norway

**Keywords:** Return to work, Sick leave, Psychotherapy, Mental health, Prognosis

## Abstract

*Purpose* Common mental disorders (CMDs) account for a large portion of sickness absence. Even after attending return to work (RTW) interventions, many patients with a CMD remain on sick leave. To identify people at risk of long-term work disability, more needs to be known about factors that predict RTW after treatment. *Methods* This was a prospective cohort study that followed 106 former patients at an RTW outpatient clinic for CMDs for 6 months after the end of treatment. Changes in work participation and mental health status between the end of treatment and the 6-month follow-up were analysed. Changes in work participation were used to identify patients with successful RTW. Patient characteristics and end-of-treatment measures of mental health status, work ability, generalized self-efficacy and expectations of future work ability, and changes in clinical outcome measures during treatment were included in logistic regression analyses to identify predictors of RTW at the 6-month follow-up. *Results* In the final model, high occupational status and higher work ability at the end of treatment predicted successful RTW at the 6-month follow-up. Further analyses showed that if the expectancy of future work ability improved or remained positive from before to the end of treatment, this was also strongly associated with RTW at the 6-month follow-up. *Conclusions* Among patients treated for CMDs, those with a low occupational status and who report lower work ability at the end of treatment are at risk of long-term disability.

## Introduction

Mental health problems account for a growing proportion of sickness absence and disability pension. The main contributors to the economic burden of reduced workdays are common mental disorders (CMDs) such as depression, anxiety and adjustment disorders [1]. In Norway, CMDs account for about one-fifth of sick leave episodes and one-third of all disability pensions [2]. Programmes and interventions tailored to facilitate return to work (RTW) after sickness absence caused by mental disorders have been developed in recent years [3], but the effects of these interventions vary [3–5]. Often, a number of participants remain on sick leave immediately after, and even 6 or 12 months after, the end of an RTW intervention [6–8].

Return to work after sickness absence is a multifactorial process, and health-related factors only partly explain individual differences [9, 10]. Many variables have been suggested as predictors of RTW, but the research is inconclusive. Older age seems to be a predictor of long-term disability among people sick-listed because of mental disorders, but the research findings vary in relation to other personal factors such as sex, educational level and marital status [10, 11]. The presence of depression and anxiety [10–12], duration and severity of depressive symptoms [13], duration of sickness absence at the onset and earlier episodes of sickness absence have all been associated with RTW [14]. A person’s own expectation of RTW is a predictor of actual RTW [7, 15], and self-efficacy—a person’s belief in being able to handle new and challenging situations—also predicts RTW [16]. In an earlier study on patients in treatment for CMDs, we found that baseline work ability, expectancy of future work ability and a history of psychiatric treatment predicted RTW at the end of treatment [17]. There might also be different predictors for RTW in different parts of the RTW process. Øyeflaten et al. found that lower educational level was a predictor of failed RTW at the 12-month but not the 3-month follow-up [6]. The same study also found that instrumental mastery-oriented coping was a positive predictor of RTW at 3 months but was a risk factor for failed RTW at 12 months.

There is insufficient knowledge about the variables that predict long-term RTW after RTW interventions. A better understanding of these factors may help identify patients at risk of failed RTW and long-term disability. In this paper, we included patients treated in an RTW programme for CMDs. We asked the following three questions: (a) Are there changes in work participation, work ability, expectations of future work ability, self-efficacy and symptoms between the end of treatment and the 6-month follow-up?; (b) Are patient characteristics and mental health status at the end of treatment associated with RTW at the 6-month follow-up?; and (c) Are improvements in symptoms, work ability, expectations of future work ability and generalized self-efficacy from before to the end of treatment associated with RTW at the 6-month follow-up?

## Methods

### Design and Treatment Setting

This was a prospective cohort study with a 6-month follow-up that studied a cohort of patients treated at an RTW outpatient clinic. This study was part of a larger study that also investigated patient characteristics before treatment [18] and predictors of RTW at end of treatment [17]. Data were collected from patients and therapists using written questionnaires, and from medical records. The RTW clinic is part of the Lovisenberg Community Mental Health Centre in Oslo and employs clinical psychologists as therapists to treat patients referred by their general practitioner (GP). To be included in the programme, a patient must have a job and be entitled to sick leave benefits. In Norway, an employee has the right to sick leave benefits equivalent to his or her full salary for a maximum of 52 weeks. After this, the person may apply for other types of benefits, which are usually at a lower level. Patients in the RTW programme can be on sick leave already or at risk of requiring sick leave (according to their GP) because of mental health problems. The programme, therefore, also has a preventive function. The treatment offered is individual, time-limited psychotherapy and/or psycho-educative courses for various problems such as depression, social phobia, panic disorder, stress and insomnia.

### Recruitment and Sample

**Fig. 1:**
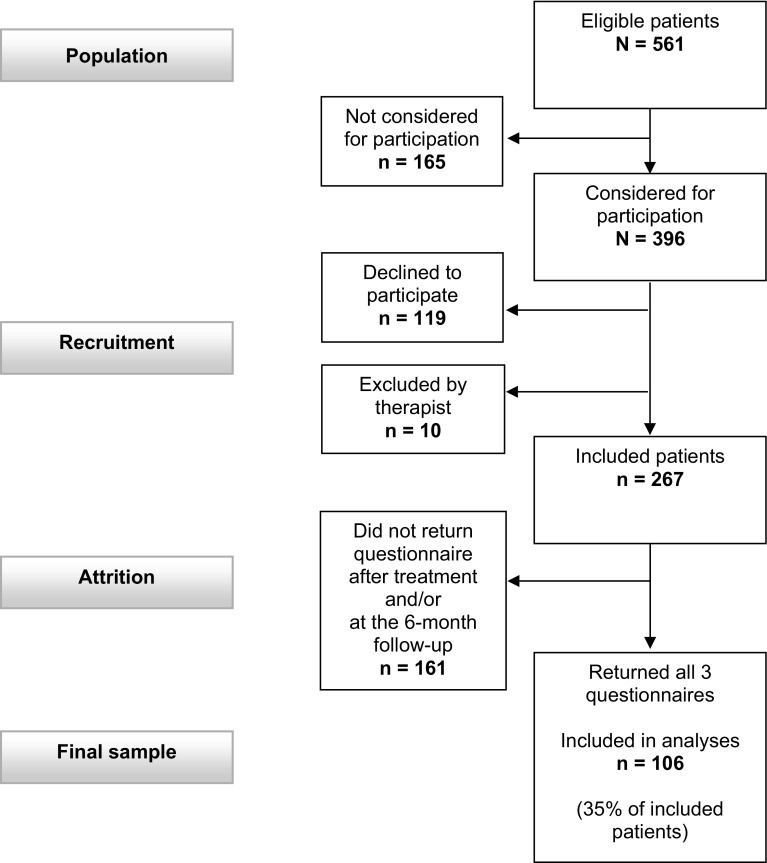
Flowchart of patient recruitment

Participants were recruited from a population of 561 patients who attended their first session at the RTW clinic during a period of ten consecutive months (16 August 2010–15 June 2011). Figure 1 shows the recruitment process. All new patients were eligible for participation in the study. One hundred and sixty-five of the eligible patients were never asked to participate, primarily because their therapist forgot to ask them to participate or because the therapist misunderstood which patients should be asked. One hundred and nineteen patients declined to participate, and ten patients were excluded. The reasons for exclusion were that the therapist considered that participation would be a burden for a patient (n = 8) or because a patient was not sufficiently fluent in the Norwegian language to complete the questionnaire (n = 2). The final sample comprised 106 patients who completed the questionnaire before and after treatment, and at the 6-month follow-up.

To investigate whether recruitment had introduced a selection bias, we examined whether there were differences between included patients (n = 267) and all other eligible patients (n = 294) (Fig. 1). Data were collected from medical records for all patients in the population (n = 561). No statistically significant differences were found for age, sex, therapist-scored symptoms and functioning, or psychiatric diagnoses. Therefore, we concluded that the included patients were representative of the population on these variables. To determine whether attrition had introduced a bias into the final sample, we examined whether there were differences between the 106 patients who returned the questionnaire at all three time points and all other included patients (n = 161). These analyses are not shown, but no statistically significant differences were found for age, sex, marital status, educational level, main diagnosis, history of psychiatric treatment, aspects of work situation causing present problem or for pretreatment measures of patent-scored symptoms, therapist-scored symptoms and functioning, work ability, expectancy of future work ability or generalized self-efficacy. We therefore concluded that the sample was representative of all included patients on these variables.

The patient characteristics at the baseline are shown in Table 1. Their mean age was 38.5 years [standard deviation (SD) = 10.0]; 74% had a college or higher university degree. Eighty-one patients (82%) could be defined as high-skilled white-collar workers, 15 (15%) as low-skilled white-collar workers and 3 (3%) as high-skilled blue-collar workers. Most patients had a CMD as their main diagnosis. Some of the patients also received secondary diagnoses, but this information was not collected systematically enough to be used in the analyses. 12 (11%) of the patients who met only for psycho-educative courses were never diagnosed, and these patients were excluded from the analyses of the diagnoses. Sixty-three patients (60%) reported that aspects of their work situation were causing the problems for which they were seeking help. Eighty-four patients (80%) scored above the clinical cut-off for psychological distress before treatment. The average duration of treatment was 36 weeks (SD = 22) and the average number of sessions was 20 (SD = 17, range 1–96). 73 (70%) patients received individual psychotherapy, 14 (13%) received group interventions only and 19 (18%) received a combination of individual psychotherapy and group interventions. The average numbers of sessions for these three groups were 18 (SD = 11), 10 (SD = 4) and 35 (SD = 17), respectively.

**Table 1:** Patient characteristics before treatment

Patient characteristic	N^a^	%
Age (years)		
18–29	21	20
30–39	43	41
40–49	26	25
50–	16	15
Sex		
Men	29	27
Women	77	73
Marital status		
Living alone	52	49
Living with partner	54	51
Education		
Comprehensive school (1–9 years)	6	6
Secondary/vocational school (10–12 years)	22	21
College degree (13–16 years)	55	52
Higher university degree (> 16 years)	23	22
Occupational status		
Low-skilled blue-collar workers	0	0
High-skilled blue-collar workers	3	3
Low-skilled white-collar workers	15	15
High-skilled white-collar workers	81	82
Main diagnosis (ICD-10)		
Depression (F32–F33)	47	50
Anxiety (F40–F42)	15	16
Adjustment disorder (F43)	20	21
Other psychiatric diagnoses	10	11
Z-diagnoses	2	2
History of psychiatric treatment		
Yes	47	44
No	59	56
Aspects of work situation causing present problem		
(1) Yes, definitely	25	24
(2) Yes, to some degree	38	36
(3) No, not really	25	24
(4) No, absolutely not	17	16

^a^N ranges from 94 to 106 because of missing data

### Measurements

The questionnaire completed by the patients covered socio-demographics (age, sex, marital status, and educational level), work situation and mental health. In the logistic regression analyses, a dichotomous variable was created for educational level (more/less or equal to 13 years of education). The therapists diagnosed each patient and completed a form containing questions about each patient’s present problems and treatment history. After the treatment, the therapist again answered questions about the patient’s present status.

#### Work Participation

Using information from the questionnaires completed by the patients and therapists, and the medical records, we constructed an index for work participation with four mutually exclusive categories: (1) *Working fully*; (2) *Working partly* (working part-time and on sick leave, receiving a social benefit or partially unemployed); (3) *Not working* (full sick leave or unemployed, and/or receiving social benefits or studying full time, including working additional hours part-time or receiving some form of social benefit); and (4) *Other* (respondents who did not fit into any of the other categories, such as being on maternity leave). The category Other included no patients before and after treatment. At the 6-month follow-up, the Other category included six patients: five were on leave from work (two on maternity leave and three on leave from work for unspecified reasons) and one had retired from work. These six patients were omitted from the analyses of changes in work participation. Patients were categorized into four groups based on the International Standard Classification of Occupations (ISCO-88) codes [19]: (1) high-skilled white-collar workers (e.g., teachers, nurses, social workers and engineers); (2) low-skilled white-collar workers (e.g., childcare workers and nursing assistants); (3) high-skilled blue-collar workers (e.g., construction workers and other skilled workers); and (4) low-skilled blue-collar workers (e.g., cleaners and other elementary occupations). In the logistic regression analysis of predictors of RTW, a dichotomous variable for occupational status was made by merging the last three alternatives.

#### RTW

Using the categories for work participation after treatment and at the 6-month follow-up, a new variable comprising five categories to define RTW was created. Moving to the Working fully category at the 6-month follow-up from any of the other categories after treatment was defined as *Full RTW*. Moving to the Working partly category from categories indicating no work participation after treatment was defined as *Partial RTW*. No change in work participation was defined as *Still working fully, Still working partly* or *Still not working*. Moving to a category indicating less work participation was defined as *Working less*. In the logistic regression analyses, a dichotomous variable for RTW was created as follows. Participants classified as having full or partial RTW were clustered together with those still working fully or partly, and their cases were defined as *successful RTW*. Those working less were clustered together with those still not working, and their cases were defined as *failed RTW*.

#### Mental Health and Psychological Variables

Symptoms of psychological distress were measured using the Clinical Outcomes in Routine Evaluation Outcome Measure (CORE-OM) [20], a self-administered questionnaire with 34 items related to the preceding week. All items are scored from “Never” (= 0) to “Almost all the time” (= 4). Total mean scores are usually multiplied by ten before being presented as a total score ranging from 0 to 40 [21]. Forms with fewer than 90% of the items completed were excluded from the analyses of the total score. An internal consistency for the CORE-OM of Cronbach’s coefficient (α) = 0.94 and a 1-week test–retest reliability of Spearman’s r = 0.90 have been reported [22]. Consistent with this, we found Cronbach’s α = 0.95 for the data at the end of treatment in this study.

The therapists diagnosed the patients according to the International Classification of Diseases 10 (ICD-10) guidelines [23], and the ICD-10 diagnoses were clustered into five categories: *Depression*, including depressive episodes (F32) and recurrent depressive episodes (F33); *Anxiety*, including phobic anxiety disorders (F40); other anxiety disorders (F41) and obsessive–compulsive disorder (F42); *Adjustment disorders* (F43); *Other psychiatric diagnoses* (e.g., substance abuse and eating disorders); and *Z-diagnoses* (reasons for contact with health services not resulting in a psychiatric diagnoses, e.g., examination). For the logistic regression analyses, three dummy variables were created, in which each of the diagnoses Depression, Anxiety and Adjustment disorder was contrasted against the other two diagnoses combined. Therapists also scored the patients on the Global Assessment of Functioning (GAF), a 100-point scale from the Diagnostic and Statistical Manual of Mental Disorders—Fourth Edition, which is used to assign a global rating of a patient’s social, occupational and psychological functioning (1 is the lowest and 100 is the highest score) [24]. Since 1998, Norwegian clinicians have used a split version of the GAF, with one scale for symptoms (GAF-S) and another scale for social functioning (GAF-F).

The work ability index (WAI) is a seven-item questionnaire used in occupational health services and research to assess work ability [25]. We used two single items from the WAI. The first item, “Assume that your work ability at its best has a value of 10 points. How many points would you give your current work ability?” is scored from 0 (= worst) to 10 (= best). A high correlation between this single item for rating work ability and the total WAI score has been reported, and this single item is often used instead of the complete instrument [26]. The second question used was “Do you believe, according to your present state of health, that you will be able to do your current job 2 years from now?” The original alternative answers in this question are: (1) “Unlikely”, (2) “Not certain” and (3) “Relatively certain”. Instead, we used the following answer alternatives: (1) “Yes, definitely”, (2) “Yes, to some degree”, (3) “No, not really” and (4) “No, absolutely not”. In the logistic regression analysis of predictors of RTW, a dichotomous variable was made by merging the first two alternatives and the last two alternatives. A dichotomous variable was also made for change on this variable: answers indicating either a change from a negative to a positive expectancy of future work ability or maintained positive expectancy of future work ability were combined, and answers indicating either a change from a positive to a negative expectancy of future work ability or a maintained negative expectancy of future work ability were combined. By providing the same distribution of individuals within the two categories, this variable to represent change from the beginning to end of treatment became identical to the dichotomous variable describing the status at the end of treatment. This showed a high stability over the time between the two measurement times.

Patients also indicated to what extent the problems they were seeking help for were caused by aspects of their work situation, using the following answer alternatives: (1) Yes, definitely, (2) Yes, to some degree, (3) No, not really and (4) No, absolutely not. In the logistic regression analysis of predictors of RTW, a dichotomous variable was made by merging the first two alternatives and the last two alternatives.

The generalized self-efficacy (GSE) scale assesses an individual’s beliefs in his or her own ability to deal with new or difficult situations [27]. Possible responses on the 10 items range from “Not at all true” (= 1) to “Exactly true” (= 4). A mean score of 1–4 is calculated for all items. The scale has been used in many research projects and typically yields an internal consistency Cronbach’s α value of 0.75–0.91 [28]. For the end of treatment data, we found a Cronbach’s α = 0.87 in this study.

#### Statistical Analyses

Data were analysed using SPSS for Windows, version 24 (IBM Corp., Armonk, NY). Differences between groups were analysed using analysis of variance for continuous and ordinal variables, and the Chi square test for categorical variables. Paired *t* tests for continuous variables and marginal homogeneity tests for categorical variables were used to identify differences from before to after the follow-up. In all analyses of differences, a significance level of *p* < 0.05 was used. Effect sizes (Cohen’s d) were calculated for changes in work ability and self-efficacy, and was defined as small (d = 0.2), medium (d = 0.5) or large (d = 0.8) [29]. We adopted Jacobson and Truax’s criterion c for establishing the clinical cut-off for the CORE-OM total score [30]. Using the SD from a Norwegian non-clinical sample [31] produced a clinical cut-off of 11.1. Using already published data from before treatment [18], the changes in clinical outcome measures during treatment were calculated. These analyses are not shown, but all changes were statistically significant. Logistic regression analysis was used to identify factors associated with RTW at the 6-month follow-up. Patient characteristics before treatment, mental health variables after treatment and changes from before to after treatment were used as independent variables. Univariable logistic regression analyses were first performed for all independent variables, with RTW as a dependant variable. Variables that had a *p* value of < 0.20 in the univariable analyses were selected for inclusion in the multivariable logistic regression analysis. Multicollinearity between the remaining independent variables was tested by checking the variance influence factor (VIF) statistics. Multicollinearity was assumed when VIF scores were > 4. A multivariable logistic regression analysis with manual backwards stepwise selection was then performed, using a *p* value of < 0.05 as the cut-off for inclusion in the final combined model (Wald statistics).

## Results

### Changes at the 6-Month Follow-Up

Changes in outcome measures between the end of treatment and the 6-month follow-up are shown in Table 2. Analysis of the changes in the group means showed significant improvements in work ability and self-efficacy. Self-assessed work ability increased by 0.74 points, yielding an effect size of d = 0.28, and GSE increased by 0.06 points, yielding an effect size of d = 0.13. There were no significant changes in self-reported symptoms or work participation. Of those working fully at the end of treatment, 48 (86%) had maintained full work participation and 8 (14%) were working less at the 6-month follow-up. Of those working partly at the end of treatment, 12 (57%) achieved full RTW, 7 (33%) had maintained partial work participation and 2 (10%) were working less at the 6-month follow-up. Of those not working at the end of treatment, 5 (25%) achieved full RTW and 1 (5%) achieved partial RTW during the follow-up, and 14 (70%) were still not working. Patients with either full (n = 17, 18%) or partial (n = 1, 1%) RTW at the 6-month follow-up, or fully (n = 48, 49%) or partially (n = 7, 7%) maintained work participation were defined as *successful RTW* (n = 73, 75%). Fourteen patients (14%) were not working at the end of treatment or the 6-month follow-up, and 10 (10%) were working less at the 6-month follow-up than at the end of treatment. These 24 (25%) cases were defined as *failed RTW*.

**Table 2:** Work participation, expectations of future work ability, work ability self-efficacy and symptoms immediately after treatment and at the 6-month follow-up

	After treatment		At the 6-month follow-up		Significance testing
	N	%	N	%	Sig.
Work participation (n = 97)					0.370^a^
Fully working	56	58	65	67	
Partially working	21	22	10	10	
Not working	20	21	22	23	
Expectations about future work ability 2 years from now (n = 60)					0.493^a^
Yes, definitely	39	65	42	70	
Yes, to some degree	14	23	7	12	
No, not really	6	10	7	12	
No, absolutely not	1	2	4	7	

	Mean	SD	Mean	SD	*t* value	Sig.
Work ability (n = 105)	6.4	2.7	7.1	2.5	− 3.003	0.003
Generalized self-efficacy (n = 105)	2.8	0.4	2.9	0.5	− 2.072	0.041
CORE-OM total (n = 105)	11.2	5.6	11.3	5.4	− 0.265	0.792
CORE-OM Anxiety (n = 105)	11.9	8.0	12.6	7.2	− 0.925	0.357
CORE-OM Depression (n = 105)	14.0	8.3	14.4	9.0	− 0.451	0.653

^a^Marginal homogeneity test, asymptotic significance (two-sided)

### Predictors of RTW

The results of the logistic regression analysis to identify factors associated with RTW are shown in Table 3. In the univariable analyses, the following variables were associated with RTW at the level of *p* < 0.20: educational level, occupational status, symptoms (CORE-OM total), symptoms (GAF-S) and functioning (GAF-F) as scored by the therapists at the end of treatment, work ability and positive expectancy of future work ability at the end of treatment. These variables were therefore considered for inclusion in the multivariable analysis. Analysis of VIF statistics showed collinearity between GAF-S and GAF-F (VIF > 4), and only GAF-F was included in the multivariable analysis. GAF-F was given precedence because a score for functioning was judged to be more relevant to the dependant variable RTW than a symptom score. Collinearity was also found between educational level and occupational status (VIF > 4), and only occupational status was included in the multivariable analysis. Occupational status was given precedence because in terms of developing interventions to support workers who are returning to work after a common mental disorder, it would be more feasible to implement an intervention according to occupational skill level rather than educational level. Backwards stepwise multivariable logic regression analysis was then performed with *p* < 0.05 as the cut-off for including independent variables in the final model. This produced a final model for predicting RTW at the 6-month follow-up, with high occupational status (odds ratio; OR 5.43, 95% CI 1.46–20.24) and higher work ability score at the end of treatment (OR 1.36, 95% CI 1.09–1.70) identified as significant predictors.

**Table 3:** Univariable and multivariable associations with RTW at the 6-month follow-up

	Univariable				Multivariable			
	OR	95% CI		*p*	OR	95% CI		*p*
		Lower	Higher			Lower	Higher	
Patient characteristics before treatment								
Sex (women)	0.72	0.25	2.07	0.547				
Age, years (older)	0.99	0.95	1.04	0.783				
Marital status (living with partner)	0.70	0.28	1.76	0.447				
Education (≥ 13 years)	3.91	1.43	10.64	0.008^d^				
Occupational status (high)	6.00	1.83	19.67	0.003^e^	5.43	1.46	20.24	0.012
History of psychiatric treatment (yes)	1.74	0.66	4.58	0.259				
Diagnosis of depression	0.86	0.30	2.44	0.778				
Diagnosis of anxiety	1.26	0.31	5.14	0.748				
Diagnosis of adjustment disorder	1.02	0.31	3.33	0.968				
Aspects of work situation causing present problem (yes)	0.70	0.27	1.85	0.471				
Mental health status at end of treatment								
Higher CORE-OM total	0.92	0.85	1.00	0.056^e^				
Higher GAF-S^a^	1.05	1.00	1.11	0.038^c^				
Higher GAF-F^b^	1.08	1.03	1.14	0.003^e^				
Higher work ability	1.40	1.14	1.72	0.002^e^	1.36	1.09	1.70	0.007
Higher generalized self-efficacy	1.18	0.41	3.37	0.763				
Positive expectancy of future work ability (yes)	15.90	2.80	90.33	0.002^e^				

Logistic regression with RTW at the 6-month follow-up as the dependent variable. Independent variables are patient characteristics before treatment and mental health status at the end of treatment. Odds ratio (OR) for RTW; an OR > 1 indicates a higher probability of successful RTW at the 6-month follow-up

N ranges from 75 to 97 because of missing data

^a^Higher score indicates fewer symptoms

^b^Higher score indicates better functioning

^c^Was not included in the multivariable analysis because of the high correlation with GAF-F

^d^Was not included in the multivariable analysis because of the high correlation with occupational status

^e^These variables were included in a backwards stepwise multivariable logistic regression with *p* < 0.05 as cut-off for including independent variables in the final model. This resulted in a final model in which high occupational status and higher work ability at the end of treatment were significantly associated with RTW at the 6-month follow-up

Additional analyses were performed to interpret these findings. Occupational status was significantly correlated with educational level (r_s_ = 0.78, *p* < 0.001). Almost all patients (97%) with a higher educational level were high-skilled white-collar workers, whereas only 24% of those with less education fell into this category. Patients with a lower educational level were predominately (67%) low-skilled white-collar workers. The variable “Are there aspects of your work situation causing the present problem?” was not associated with occupational status or educational level. Individuals with a high occupational status received, on average, five more sessions than individuals with a lower occupational status, but this difference was not significant (*p* = 0.208). Neither the number of sessions nor the type of treatment predicted RTW at the 6-month follow-up.

### Secondary Outcome Measures and RTW

Univariable logistic regression was performed with RTW at the 6-month follow-up as a dependent variable and changes in other outcome measures as independent variables (Table 4). Improved GAF-F, improved work ability and improved expectancy of future work ability were associated with RTW at a level of *p* < 0.20. These three variables were therefore included in a backwards stepwise multivariable logistic regression with *p* < 0.05 as the cut-off for including independent variables in the final model. This produced a model in which only improved expectancy of future work ability was significantly associated with RTW at the 6-month follow-up (OR 15.90, 95% CI 2.80–90.33).

**Table 4:** Logistic regression analyses to investigate whether changes in secondary outcome measures between before and after treatment are associated with RTW at the 6-month follow-up

	Univariable associations				Multivariable associations			
	OR	95% CI		*p*	OR	95% CI		*p*
		Lower	Higher			Lower	Higher	
Changes between before and after treatment								
Improved CORE-OM	1.07	0.97	1.18	0.210				
Improved GAF-S	1.02	0.96	1.08	0.515				
Improved GAF-F	1.04	0.98	1.10	0.168^b^				
Improved work ability	1.15	0.96	1.38	0.131^b^				
Improved generalized self-efficacy	0.60	0.24	1.56	0.297				
Improved expectation of future work ability^a^	15.90	2.80	90.33	0.002^b^	15.90	2.80	90.33	0.002

The dependent variable is RTW; an odds ratio (OR) for RTW of > 1 indicates a higher probability of successful RTW at the 6-month follow-up

N ranges from 70 to 97 because of missing data

^a^On this variable, being a case indicates either a change from negative to positive expectancy of future workability, or a maintained positive expectancy of future workability

^b^These variables were included in a backwards stepwise multivariable logistic regression with *p* < 0.05 as the cut-off for including independent variables in the final model. This resulted in a final model in which only improved expectancy of future work ability between before and after treatment was significantly associated with RTW at the 6-month follow-up

## Discussion

In this study, we tried to identify predictors of RTW at 6 months after the end of treatment, among patients treated for CMDs. The main finding is that a high occupational status and higher work ability after treatment predicted successful RTW at the 6-month follow-up. We also found that if the expectancy of future work ability improved or remained positive from before to after treatment, this was also associated with successful RTW at the 6-month follow-up.

### Changes at the 6-Month Follow-Up

We found that there was no significant change in work participation between the end of the treatment and the 6-month follow-up, even if full work participation had increased from 58 to 67%. Other studies have reported increases in full RTW after treatment but have not always tested these changes statistically. Lagerveld et al. [32] reported that the percentage of people achieving full RTW increased in the intervention group from 73% at the 6-month follow-up to 96% at 12 months. We found that self-assessed work ability had improved at the 6-month follow-up, yielding a small effect size. This measure has been shown to be a strong predictor of future sick leave and increased risk of early retirement from the labour market [33]. In our study, the self-efficacy score improved slightly and there were no significant changes in self-reported symptoms.

### Predictors of RTW

In the final model, we found that high occupational status predicted successful RTW at the 6-month follow-up. People in higher status jobs are known to experience more autonomy and control in their work [34, 35]. Because of this, we wonder if it was possible to make more adjustments in their work situation, which might compensate for symptoms and reduced work ability. Six of ten patients confirmed that there were aspects of their work situation causing the present problem. This indicates that working environment influenced the patients’ health and functioning, but at the same time this variable was not associated with RTW. We also found no association between this variable and occupational status. There might be other ways in which occupational status is indirectly associated with the rate of RTW. In our study, high occupational status was related to high educational level. A higher educational level is associated with health-promoting behaviour [36, 37] and better prognosis of illness [38, 39]. In our study, high educational level was associated with RTW in the univariable analyses. This is interesting because educational level did not predict RTW at the end of treatment in our earlier study of patients in treatment for CMDs [17]. However, this is consistent with the findings of Øyeflaten et al. [6], who reported that lower educational level predicted failed long-term, but not short-term, RTW. We conclude that it is not clear why high occupational status predicted RTW at the 6-month follow-up. There are several possible explanations, including differences in both individual workers and their working situations. These are important aspects of understanding the RTW process and need further investigation.

In the final model, higher work ability at the end of treatment predicted successful RTW at the 6-month follow-up. This is consistent with earlier research showing that self-assessed work ability predicts future sickness absence [26, 40], disability leave [40, 41] and early retirement from the labour market [33]. Using the same single item from the WAI to measure self-assessed work ability as we did in this study, Sell et al. found that a one-point decrease in this item at the baseline was associated with a 15% increased risk of long-term sickness absence and a 33% increased risk of early retirement [33]. Self-assessed work ability has also been shown to be associated with symptoms of CMD [42] and both mental and physical health problems [43]. The predictive power of self-assessed work ability may therefore reflect an association with symptoms. However, in our study, self-assessed work ability also remained a significant predictor of RTW after controlling for the levels of symptoms.

In our univariable analyses, expectancy of future work ability was a strong predictor of RTW. This is consistent with recent reports that found that expectations of future work ability and RTW predict actual RTW [7, 15]. However, in the final model, which controlled for occupational status, patient- and therapist-scored symptoms and work ability, expectancy of future work ability no longer predicted RTW. When investigating how improvements in clinical outcome measures achieved during treatment were associated with RTW, we found that, if the expectation of future work ability improved or remained positive, this was a strong predictor of RTW at the 6-month follow-up. It seems that expectancy of future work ability is an important factor to understanding RTW, but interactions with other variables need to be investigated further. Symptom level, as reported by the patient at the end of treatment, was not a significant predictor of RTW, whereas a lower symptom score and higher functioning, as reported by the therapists, predicted successful RTW in the univariable analyses. It is unclear how this finding should be interpreted, but it may be clinically useful if it could be confirmed that a therapist’s assessment of the patient’s symptoms and functioning at the end of treatment can predict long-term RTW.

### Strengths and Limitations

One strength of this study is that it included variables relating to the patients’ characteristics, psychological variables and clinical status. This made it possible to investigate both demographic and clinical variables as predictors of RTW. We also used measurements that are routinely used in clinical practice to measure symptoms and functioning. This means that the same information would be available to clinicians who, near the end of treatment, must decide whether to terminate the therapy with a patient even if RTW is not yet achieved. One limitation of the study is that there are other potential predictors that were not included, for example, the working environment. We also did not collect any information about interventions during the follow-up and, therefore, we could not control whether these factors had any influence on the RTW outcome. In this study, we defined RTW as both returning to work after sick leave and when maintaining work participation when at risk of taking sick leave. In many clinical settings, therapists would be treating patients from both groups. However, it cannot be ruled out whether different mechanisms influence RTW after sick leave and when maintaining work participation when at risk of taking sick leave. Because of the sample size, it was not possible for us to investigate potential subgroup differences in this study. Another limitation is the low number of participants included in the logistic regression analyses in relation to the number of potential predictors. A larger sample size would have made the final model more reliable and may have produced more significant findings. Therefore, the non-significant findings may not be generalizable. We found that improved expectancy of future work ability was significantly associated with RTW. The OR of 15.90 and 95% CI of 2.80–90.33 meant that the variance was very large. This finding therefore needs to be interpreted with some caution.

Another possible weakness of this study is that the selection method might have introduced a bias into the patient selection. However, we were able to collect some data for the entire population and found that age, sex, GAF scores and diagnoses did not differ significantly between the included patients and the whole population. In this study, attrition was high, which suggests that self-selection might have introduced a bias into the final sample. However, analysis showed that there were no significant differences in patient characteristics or mental health status before treatment between dropouts and the final sample. We therefore conclude that, for these variables, the sample did not differ significantly from all included patients.

### Practical Implications and Further Research

A number of patients with CMDs will not achieve RTW during a 6-month follow-up after treatment has ended. When planning RTW interventions, efforts should be made to identify individuals at risk of long-term disability after the intervention. People with lower occupational status and who, at the end of treatment, report lower work ability are at risk of long-term disability. This group might benefit from a prolonged intervention or an adjusted intervention. Future research should investigate possible differences in the predictors of RTW after sick leave and maintained work participation in patients at risk of requiring sick leave. Future research should also investigate the role of occupational status in determining long-term RTW.
